# Severe Neutropenia and Vitamin B12 Deficiency in a Patient With Recreational Nitrous Oxide Use and History of Polysubstance Use: A Case Report

**DOI:** 10.7759/cureus.91279

**Published:** 2025-08-30

**Authors:** Lindsay A Turmelle, Carl Chotas

**Affiliations:** 1 Pediatrics, Edward Via College of Osteopathic Medicine, Blacksburg, USA; 2 Hospital Medicine, Johnston Memorial Hospital, Edward Via College of Osteopathic Medicine, Abingdon, USA

**Keywords:** neurological complications, nitrous oxide toxicity, pancytopenia, recreational drug use, vitamin b12 deficiency

## Abstract

Nitrous oxide, commonly known as "laughing gas," has been widely used as a medical and dental sedative for centuries. Despite its recognized therapeutic applications, there has been a concerning rise in its recreational use, particularly among young adults. This trend has led to an increase in associated toxicities, including neurological, hematological, and psychiatric complications.

This is a case report of a young adult presenting with neurological and hematological symptoms following chronic recreational nitrous oxide use. The patient exhibited diffuse abdominal pain, vomiting, nausea, confusion, and radicular pain in her upper extremities upon initial exam, which prompted an extensive diagnostic evaluation. These findings evolved during her hospital course, including the development of depressive symptoms, insomnia, and ataxia. Laboratory findings revealed evidence of severe neutropenia, pancytopenia, and vitamin B12 deficiency, supporting a diagnosis of nitrous oxide-induced neurotoxicity. Management included cessation of nitrous oxide exposure, vitamin B12 supplementation, psychiatric intervention, and supportive therapy, leading to gradual symptom improvement, which will hopefully continue in the outpatient setting.

This case highlights the growing public health concern surrounding nitrous oxide abuse. Its recreational use can result in severe and potentially irreversible neurological impairment, often linked to its association with vitamin B12 deficiency. Increased awareness among healthcare providers is crucial for early recognition and intervention. Additionally, preventive measures, including public education and regulatory policies, may help curb the rising trend of nitrous oxide abuse. It is an emerging public health issue with significant consequences, and this case highlights the importance of early diagnosis and treatment, as well as the need for increased awareness to mitigate long-term complications.

## Introduction

Nitrous oxide, commonly known as “laughing gas, “whippets,” or “whip-its”, has been used as an inhaled sedative in medical and dental settings dating back to the 1800s [1], and is still widely used today despite the emergence of newer agents [2]. Medical professionals may use it to sedate patients who are having minor medical procedures, and it commonly serves as an adjunct to general anesthesia due to its anxiolytic and pain-relieving properties [1-3]. It is generally mixed with 30-70% oxygen, though nitrous oxide rarely surpasses 40%, to prevent the development of adverse effects in patients [4,5]. Recreationally, however, it is often unmixed, and if 100% nitrous oxide is inhaled, it enters the alveoli, displaces oxygen in the lungs, and can lead to hypoxia, adverse sequelae, or death [4,5].

Although its adverse and toxic effects are well known, there has been an increase in the prevalence of its recreational use in the United States, particularly among young adults [5]. The Global Drug Survey, a study of more than 32,000 participants in 22 countries, found that nitrous oxide use had increased from 10% in 2015 to 20% in 2021 [6]. Its use was highest among people 16-24 years old in the United Kingdom, though this estimate of prevalence could be limited due to the voluntary nature of the conducted survey [6]. This same survey revealed that nitrous oxide is the second most common drug of abuse among 16-24-year-olds in the United Kingdom, with rising prevalence possibly related to isolation endured during the COVID-19 pandemic [6,7]. In the United States, inhalant abuse is most common among adolescents aged 12 to 17 [8]. A 2019 report by the Substance Abuse and Mental Health Services Administration (SAMHSA) noted that 3.0% of adolescents and 1.7% of adults aged 18-25 reported using inhalants within the past year [7,8]. It evades surveillance because it is not classified as a controlled substance and is widely available to order online or purchase over the counter. Its users are able to get a legal “high” from inhaling nitrous oxide from pressurized canisters [7,9]. Nitrous oxide is recreationally used for its immediate induction of euphoria, dissociation, hallucinations, and anti-anxiety effects, which typically last a few minutes [10]. Short-term side effects of misuse include slurred speech, dizziness, headache, drowsiness, and lack of coordination [10], while significant long-term health risks include neurologic, hematologic, and psychiatric illness [7].

Nitrous oxide exerts its toxic effects by irreversibly oxidizing the cobalt ion of vitamin B12, rendering it inactive [2]. Vitamin B12 serves as a coenzyme in essential metabolic pathways, including methylation reactions critical for DNA synthesis and myelin maintenance [11]. Its inactivation leads to elevated homocysteine and methylmalonic acid levels, impairing cellular replication and disrupting myelin integrity, which explains the wide range of symptoms that patients may present in the setting of a B12 deficiency [11]. Hematologic manifestations include megaloblastic anemia and pancytopenia, which can increase susceptibility to infection when white blood cells are affected [11]. While a range of neurologic and psychiatric symptoms may present with deficiency, chronic nitrous oxide use is often seen with concurrent psychiatric illness [7,12]. Previous cases have been published regarding nitrous oxide-induced B12 deficiency causing subacute combined degeneration of the spinal cord, leading to sensory deficits, ataxia, and spasticity, as well as psychiatric disturbances such as depression and cognitive impairment [4]. MRI findings from another study have shown subacute combined degeneration of the spinal cord in 68% of patients with daily nitrous oxide abuse, but this finding on imaging need not be present for diagnosis [4]. One case report describes two young patients with histories of regular whippet use for three years, who both presented with myelopathy secondary to vitamin B12 deficiency [2]. They experienced various neurological symptoms involving progressive stiffness, tingling, weakness, and swelling in the legs [2]. Subjects of these case reports depict positive patient response to abstinence from nitrous oxide use, vitamin B12 supplementation, and physical therapy [2]. Despite the well-documented adverse effects of chronic misuse, it is important to note that pediatric patients, with no suspected vitamin B12 deficiency, who are exposed to nitrous oxide for several hours during surgery, do not develop signs of megaloblastic anemia, pancytopenia, thrombocytopenia, or leukopenia [13] likely due to appropriate dosing and mixture with oxygen. Thus, it is chronic, recreational use that poses the most harm to vulnerable populations.

This report depicts a 21-year-old patient with a history of polysubstance use disorder who was admitted to the hospital with severe neutropenia and subsequently diagnosed with severe vitamin B12 deficiency. This patient’s vitamin deficiency was tied to daily nitrous oxide use for several years prior to admission. Historically, reviews of literature and case reports have discussed the increased need to screen for B12 deficiencies in young patients presenting with neurological symptoms due to the growing prevalence of nitrous oxide abuse. However, long-term sequelae of nitrous oxide abuse are still underreported, and further research is necessary to explore how best to provide adequate education and intervention to these patients. This report will discuss how early recognition and intervention are essential to preventing or reducing irreversible neurological damage and risk of infection in nitrous oxide users.

## Case presentation

A 21-year-old Caucasian female with a past medical history of anxiety, depression, and polysubstance use disorder presented to the emergency department with worsening diffuse abdominal pain that radiated to her back, nausea, vomiting, and significant suprapubic pain with associated urinary incontinence and dysuria. The patient reported several months of nausea and vomiting whenever she tried to eat, resulting in significant weight loss with worsening weakness, depressive symptoms, and cognitive dysfunction. She was not taking any prescribed medications. Known allergies included fish-derived products. Her past medical history was notable for polysubstance abuse of fentanyl, marijuana, and cocaine, of which she denied use for several months prior to admission. She endorsed the current use of kratom, which she purchased from the smoke and vape shop where she was employed, to treat her pain. She denied tobacco use but did vape nicotine. Additionally, she used nitrous oxide, or “Whip-Its,” multiple times a day, with her last use being 3 hours prior to presentation to the emergency department. She reported some exercise and consumed a normal diet with no dietary restrictions prior to her symptoms. Social support consisted of her partner, with whom she lived, her mother, and her stepfather.

A thorough initial workup in the emergency department was completed. Vitals obtained in the emergency department revealed a temperature of 98.4°F, a heart rate of 87 bpm, and a blood pressure of 105/59 mmHg with an SpO_2_ of 94% on room air. Physical exam revealed reproducible abdominal tenderness in the bilateral upper quadrants and left lower quadrant with palpation. Electrocardiogram (EKG) revealed normal sinus rhythm with a heart rate of 72 bpm and early repolarization, but was otherwise unrevealing. Chest X-ray revealed no acute processes. A urinalysis was also obtained.

Advanced imaging, including a computed tomography (CT) of the abdomen and pelvis with contrast, showed no acute intra-abdominal processes, but did reveal chronic hepatic steatosis and findings suggestive of pelvic congestion syndrome. This was supported by lab values that showed elevated total bilirubin, which raised concerns for an acute obstructive hepatopathy or an acute gallbladder process. However, a subsequent abdominal ultrasound was unremarkable with a normal hepatobiliary system.

Consideration was also given to acute hemolytic processes because a complete blood count (CBC) revealed pancytopenia, specifically severe neutropenia. This neutropenia was a new finding compared to a CBC that was completed in an outpatient setting two months prior to admission. Her elevated albumin could suggest dehydration, but nephropathy could be included in the differential. Erythrocyte sedimentation rate (ESR) and C-reactive protein (CRP) were within normal limits, which demonstrated that an acute inflammatory or autoimmune process was unlikely. It was initially unclear as to why this patient was acutely immunocompromised. Furthermore, blood and urine cultures were ordered in the emergency department. The patient was started on empiric intravenous (IV) ceftriaxone, metronidazole, and doxycycline for gastrointestinal, genitourinary, and dental infection coverage, respectively, as well as IV pantoprazole twice a day for suspected peptic ulcer disease and known gastroesophageal reflux disease (GERD). IV metoclopramide three times a day was initiated for her recurrent nausea and vomiting concerning for gastroparesis. IV fluid resuscitation was initiated for dehydration, as well as ondansetron for nausea.

The patient was admitted for failure to thrive secondary to recurrent nausea and vomiting, suspected peptic ulcer disease, new-onset neutropenia, thrombocytopenia, biliary colic, and urinary tract infection. Upon admission, the patient was a cachectic, ill-appearing young woman who was fatigued but alert and in no acute distress. Multiple dental caries were visible bilaterally, but there were no signs of an obvious dental infection. She had a soft, non-distended abdomen with marked epigastric and suprapubic pain on palpation, though she displayed no peritoneal signs. She denied any history of alcohol use. An IV thiamine infusion was started, and Gastroenterology was consulted, who recommended outpatient esophagogastroduodenoscopy (EGD) once there was resolution of her pancytopenia.

On day 1 of admission, a repeat comprehensive metabolic panel (CMP) revealed resolution of her hyperbilirubinemia. Repeat CBC revealed worsening pancytopenia, though this may have been dilutional, given efforts for rehydration. Beta-human chorionic gonadotropin (βHCG) was negative. Folate and thyroid-stimulating hormone (TSH) levels were within normal limits. Hepatopathy was still considered due to a mildly elevated international normalized ratio (INR). An acute infectious process was included in the differential due to a mildly elevated procalcitonin, though she remained afebrile without signs of worsening infection. Urine culture was negative for growth. HIV testing was negative. A work-up for anemia was remarkable for iron deficiency anemia and severe vitamin B12 deficiency of <50 pg/mL (normal 200-1000 pg/mL). The differential diagnoses that were considered with vitamin B12 deficiency included malnutrition, pernicious anemia, gastritis, alcoholism, celiac disease, Crohn’s disease, and nitrous oxide abuse. Because of severe deficiency, the care team managed her condition with intramuscular (IM) injections of vitamin B12 daily. 

On day 2 of admission, she was started on vitamin B12 supplementation via IM injection. The patient reported ongoing bilateral radicular arm pain, which was not adequately controlled with as-needed hydrocodone-acetaminophen. She was instead started on gabapentin twice a day for nerve pain.

On day 3 of admission, there was mild improvement in her symptoms, and her appetite returned. She began experiencing involuntary shaking of her upper extremities and requested a benzodiazepine. Due to her previous finding of severe vitamin B12 deficiency, her proton pump inhibitor was discontinued, and she was started on an H_2_ blocker to increase her absorption of vitamin B12. Diazepam was started for her tremors and anxiety. At this time, the patient was counseled about discontinuing nitrous oxide use, and she indicated a plan to begin abstinence after discharge.

On day 4 of admission, the patient noted new difficulty with ambulation and increased depressive symptoms. She continued to have persistent abdominal and flank pain despite analgesics. The care team consulted physical and occupational therapy, to which the patient responded well. To rule out any extraneous causes for neurologic changes, a brain magnetic resonance imaging (MRI) was ordered. This was negative for acute processes. Psychiatry was also consulted, and they recommended starting duloxetine and mirtazapine to aid with sleep, depressive symptoms, and to provide possible neuropathy benefit. The patient continued to receive daily IM vitamin B12.

The patient was discharged on day 5 of admission. She had improvement in symptoms, and vitamin B12 levels had returned to the normal range. Outpatient discharge plans included close-interval follow-up with her primary care physician as well as recommendations to see gastroenterology, psychiatry, and hematology, as needed. She was discharged on duloxetine 20 mg twice daily, famotidine 20 mg twice daily, mirtazapine 15 mg daily at bedtime, and ondansetron 4 mg every 8 hours as needed for nausea for up to seven days. Notably, she was also given a three-day course of hydrocodone-acetaminophen 5-325 mg to take every 4 hours as needed and intranasal naloxone as standard protocol for unintentional opioid overdose. Importantly, instructions for continuous vitamin B12 supplementation were discussed with the patient. Her recommended course was 1 mg IM daily for two more days after discharge, then 1 mg IM once a week for 28 days, then 1 mg IM every 30 days. The patient was motivated to abstain from nitrous oxide and other substance use after discharge.

## Discussion

The proposed pathophysiology for this case is severe neutropenia and severe vitamin B12 deficiency secondary to chronic nitrous oxide abuse. In the absence of additional, significant medical and social history findings, her confusion, weakness, generalized fatigue, and pancytopenia were likely secondary to chronic daily nitrous oxide use.

This case illustrates the significant and underrecognized consequences of chronic nitrous oxide abuse, particularly its impact on hematologic and neurologic systems. The patient's pancytopenia, severe neutropenia, progressive weakness, cognitive dysfunction, and depressive symptoms were in alignment with severe, chronic B12 deficiency, as demonstrated in previous research [1,2,7,11,12]. While her history of peptic ulcer disease and proton pump inhibitor medication use could have contributed to her vitamin B12 deficiency, the severity of her lab findings more strongly suggested nitrous oxide abuse as the culprit. This case highlights the importance of taking a thorough social history to accurately assess a patient’s substance use status and how that may influence the differential diagnosis of young patients presenting with unexplained neurologic and hematologic abnormalities.

This case aligns with prior reports detailing nitrous oxide-related myelopathy and hematologic abnormalities, many of which involved young adults with histories of polysubstance use. Chronic substance use often leads to irreversible neurological damage. However, early recognition and intervention, as seen in this patient, may lead to substantial recovery. The patient’s improvement in B12 levels following supplementation shows the reversibility of symptoms when treatment is initiated promptly. Previous case studies that demonstrated success with physical therapy and occupational therapy among patients who had experienced neurotoxicity symptoms associated with B12 deficiency influenced the medical decision-making in this case [2]. However, the patient’s persistent cognitive and depressive symptoms prior to discharge highlight the potential for prolonged recovery and the need for continued outpatient management. The reciprocal nature between nitrous oxide abuse, vitamin B12 deficiency, chronic pain, and depression makes this case of particular importance in furthering multidisciplinary interventions for these patients.

Despite increasing reports of nitrous oxide toxicity, recreational use remains largely underregulated and difficult to monitor due to its legal status and widespread availability. The rise in nitrous oxide abuse, particularly among young adults, highlights the growing need for heightened awareness and screening in clinical settings. Early identification of vitamin B12 deficiency in at-risk populations is essential to prevent long-term neurological impairment, and more research must be completed to fully understand the possible irreversible damage and long-term effects. Furthermore, comprehensive intervention strategies, including patient education, psychiatric support, and substance use disorder treatment, are critical to addressing the root causes of nitrous oxide abuse.

## Conclusions

Ultimately, this case emphasizes the importance of recognizing nitrous oxide abuse as a growing public health concern. Clinicians should maintain a high index of suspicion for vitamin B12 deficiency in patients with neurological symptoms, unexplained pancytopenia, or a history of inhalant use. Future research should explore effective harm reduction strategies and the long-term outcomes of nitrous oxide-related neurotoxicity to improve patient care and public health initiatives. Given the rising prevalence of nitrous oxide abuse, more research is needed to develop effective educational, preventative, and treatment strategies to mitigate long-term complications in vulnerable populations.

## References

[1] Garakani A, Jaffe RJ, Savla D, Welch AK, Protin CA, Bryson EO, McDowell DM (2016). Neurologic, psychiatric, and other medical manifestations of nitrous oxide abuse: A systematic review of the case literature. Am J Addict.

[2] Barmak F, Numan J, Shabih M, Nolte J, Adams J, Ferguson P, Inam SH (2024). Nitrous oxide-induced vitamin B12 deficiency and myelopathy in whippets abusers: A report of two cases. Cureus.

[3] (2025). Nitrous oxide - Alcohol and Drug Foundation. https://adf.org.au/drug-facts/nitrous-oxide/.

[4] (2023). ToxTalks: Substances of Use and Misuse 
Nitrous Oxide. https://med.virginia.edu/toxicology/wp-content/uploads/sites/268/2023/08/Aug23-SAM-NitrousOxide.pdf.

[5] Sedore S (2023). Let’s “Whippit" away: Nitrous oxide misuse and its complications. Am J Psychiatry Resid J.

[6] Zaloum SA, Mair D, Paris A, Smith LJ, Patyjewicz M, Onen BL, Noyce AJ (2025). Tackling the growing burden of nitrous oxide-induced public health harms. Lancet Public Health.

[7] Gardin TM, Yang A, Moeller JJ, Quraishi IH (2023). Subacute combined degeneration of the spinal cord in a patient with nitrous oxide use and autoimmune atrophic gastritis. BMJ Case Rep.

[8] (2025). Nitrous Oxide Misuse and Abuse | Toxicology Section. https://www.acep.org/toxicology/newsroom/jun2021/nitrous-oxide-misuse-and-abuse.

[9] Dharaiya D (2025). Dharaiya D, Silverstone L, Sharma R, et al. Nitrous Oxide Toxicity. Reference Article. https://radiopaedia.org/articles/30559.

[10] (2025). Nitrous Oxide Abuse: Side Effects & Treatment for Whippets. American Addiction Centers. https://americanaddictioncenters.org/inhalant-abuse/nitrous-oxide-whippets.

[11] Ankar A, Kumar A (2025). Vitamin B12 deficiency. StatPearls [Internet].

[12] Kao A, Malik S (2022). A unique case of whippets-induced B12 deficiency manifesting as a subacute Brown-Sequard spinal syndrome (P1-1.Virtual). Neurology.

[13] Duma A, Cartmill C, Blood J, Sharma A, Kharasch ED, Nagele P (2015). The hematological effects of nitrous oxide anesthesia in pediatric patients. Anesth Analg.

